# Mechanical Responses of a Single Myelin Layer: A Molecular Simulation Study

**DOI:** 10.3390/biom13101525

**Published:** 2023-10-14

**Authors:** Fairuz Maliha, Ashfaq Adnan

**Affiliations:** Department of Mechanical and Aerospace Engineering, The University of Texas at Arlington, Arlington, TX 76019, USA; fxm7560@mavs.uta.edu

**Keywords:** molecular dynamics, myelin sheath, neuron cell, traumatic brain injury, lipid bilayer

## Abstract

The myelin sheath provides insulation to the brain’s neuron cells, which aids in signal transmission and communication with the body. Degenerated myelin hampers the connection between the glial cells, which are the front row responders during traumatic brain injury mitigation. Thus, the structural integrity of the myelin layer is critical for protecting the brain tissue from traumatic injury. At the molecular level, myelin consists of a lipid bilayer, myelin basic proteins (MBP), proteolipid proteins (PLP), water and ions. Structurally, the myelin sheath is formed by repeatedly wrapping forty or more myelin layers around an axon. Here, we have used molecular dynamic simulations to model and capture the tensile response of a single myelin layer. An openly available molecular dynamic solver, LAMMPS, was used to conduct the simulations. The interatomic potentials for the interacting atoms and molecules were defined using CHARMM force fields. Following a standard equilibration process, the molecular model was stretched uniaxially at a deformation rate of 5 Å/ps. We observed that, at around 10% applied strain, the myelin started to cohesively fail via flaw formation inside the bilayers. Further stretching led to a continued expansion of the defect inside the bilayer, both radially and transversely. This study provides the cellular-level mechanisms of myelin damage due to mechanical load.

## 1. Introduction

Myelin is a long sheet-like structure that primarily acts as a protective covering around the neuron cells. It promotes signal transmission by reducing reaction time. As such, myelin is an important cellular element of neuron cells. Earlier studies suggest that myelin is formed from two different types of cells: Schwann cells in the peripheral nervous system (PNS) and oligodendrocytes in the central nervous system (CNS) [1,2,3]. In principle, myelin is formed by folding layers of lipid membranes in a spiral pattern around the axon [1,2,3]. In addition, it has been observed from past research that forty or more lipid bilayers typically stack up to form the myelin sheath [2]. The internal structure of myelin is primarily made of 70%–85% lipid membranes [4], and the remaining fraction comprises two major types of proteins, namely, myelin basic proteins (MBP) and proteolipid proteins (PLP) [5]. In the central nervous system, myelinated axons actively interact and intertwine with adjacent neuron cells and glial cells such as oligodendrocytes [6,7].

It has been reported in the literature on experimental and clinical traumatic brain injury (TBI) that the myelin sheath may become damaged and undergo chronic degradation (thickness reduction) during a post-traumatic phase after injury [8,9,10,11,12,13,14,15,16,17,18]. Progressive dissociation and damage to the myelin layers around axons have been extensively observed in in vivo studies on mice brains, even in cases of mild traumatic brain injury (TBI) [13,14,15,16,17,18]. The disintegration of myelin layers within the brain’s white matter are frequently seen from 6 weeks to 3 months after TBIs [15,16]. Stretch injuries in axons appear to trigger the breakdown of myelin basic protein (MBP) and lead to demyelination [17]. In mice, even after 12 months following the injury, mild TBIs can persistently alter the integrity of the brain’s white matter [18]. Taib et al. [15] pointed out that such irregularities in myelin within the white matter can lead to structural fragmentation, the loosening of the myelin layers or the separation of the myelin layers from the axon. These findings imply that the structural dissociation of myelin has a direct connection with TBI. However, the fundamental biomechanisms behind myelin dissociation are not well-understood. This is probably because myelin biomechanics relate to structural, physiological and biological interactions of myelin with adjacent cellular entities in the brain.

Several studies have been carried out in the past to understand the mechano-physiological behavior of lipid bilayers, the primary structural component of the myelin sheath. It was observed that the rupture strength of the bilayers is influenced by the presence of unsaturated bonds in the hydrocarbon chain. Under tensile loading at a rate of approximately 0.1 Mn/m/s, the failure of unsaturated mono- and di-mono phosphatidylcholine (PC) bilayers was observed at the same magnitude (~10 Mn/m). This critical value dropped to ~5 Mn/m when the number of unsaturated bonds increased [19]. In addition, amyloid-forming proteins compromise the strength of bilayers by reducing the Young’s modulus (E) and adhesive properties [20]. A study on supported lipid bilayers (SLBs) portrayed the importance of lipid phases in mechanical responses by comparing the stiffness at gel phases (E = 28.1 MPa) and liquid phases (E = 19.3 MPa) [21]. A study carried out on the composition of the bilayers presented the influence of the system’s composition on the elasticity of the bilayers [22]. The bending and elastic moduli of the lipid bilayers have been studied to understand the impact of system size and individual lipid composition in the system [23,24]. To illustrate the impact of lipid type on membrane deformation, mechanisms of pore formation and the sealing process during nanoparticle penetration were studied [25]. In another study, a non-equilibrium molecular simulation was conducted to capture the effect of the oscillating strain rate on the expansion and contraction moduli of a pure DMPC bilayer [26]. A few other studies have focused on the deformation of the lipid bilayer from a biological perspective [27,28,29,30,31,32,33]. Although these studies reveal mechanobiological insights on lipid biomechanics, the mechanisms are not directly relatable to myelin biomechanics.

Few other studies have focused on the biological aspects of how myelin interacts with other components [13,34,35,36,37,38,39]. In a recent study, the interaction of myelin basic protein (MBP) in varying concentrations with the plasma membrane was studied using the Langmuir–Blodgett (LB) membrane method with AFM [34]. The bending modulus of myelin with varying levels of cholesterol was studied to understand the pathology of Alzheimer’s disease (AD) [35]. Using a finite element approach and coarse grain approach, the mechanoporation of myelin, axolemma and the node of Ranvier was studied for different strain rates, and it was observed that the failure strain was higher for myelin compared to unmyelinated axolemma and the node of Ranvier. Nevertheless, in this study the myelin models were built without the major protein classes, which was believed to influence the overall mechanical behavior of myelin [39]. With scant evidence on the mechanical response, it is difficult to understand the threshold of myelin deformation in its exposure to varying levels of cellular trauma.

Thus, it is evident that a quantitative understanding of myelin biomechanics, purely due to mechanical loading, is still lacking. To elucidate how mechanical forces affect myelin deformation and damage, first we modeled a representative volume element of a single layer of myelin sheath using the CHARMM-GUI membrane builder. Then, we analyzed the effect of tensile mechanical deformation on myelin structure. The tensile stress–strain response of myelin was generated and the mechanisms of myelin failure were discussed.

## 2. Materials and Methods

**Figure 1:**
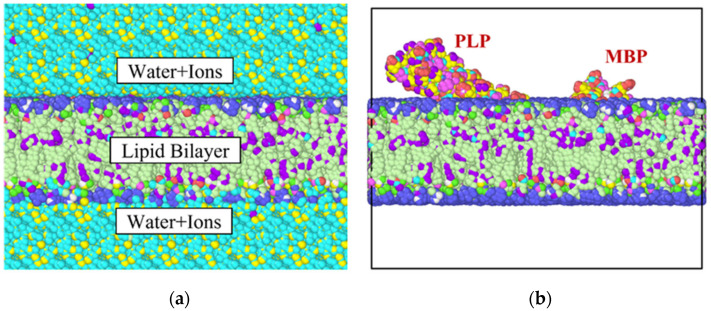
(**a**) Energy-minimized molecular structure of the representative volume element. The top and the bottom layers are occupied by water and ions and the middle layer is the lipid bilayer with proteins embedded in it. (**b**) Molecular structure of the system without water or ions (for clarity).

The molecular structure of myelin consists of a lipid bilayer, myelin basic protein, proteolipid protein (PLP), water and ions [40,41,42]. Accordingly, our simulation model comprised two types of protein and a lipid bilayer (Figure 1a,b). The simulation box dimensions were 150 × 150 × 120 Å. Microscopy studies in the past [43] have revealed that the average thickness of the myelin sheath varies between 100 and 400 nm and that a single myelin layer is approximately 10 to 20 nm thick. The percentage composition of the simulation model by constituents have been tabulated in Table 1. Thus, the following dimensions were determined for the representative elements for the myelin sheath:

Myelin thickness: 120 Å;

Lipid bilayer thickness: 40 Å;

Water box thickness (top): 40 Å;

Water box thickness (bottom): 40 Å.

The representative volume element for the simulation was developed using the CHARMM-GUI input generator [44]. The structure models of each of the constituent proteins are freely available in molecular database sites such as the RCSB PDB protein bank and the UniProt protein sequence database. To build our simulation model, we extracted the structure of MBP (4BVM, [45]) and PLP (1WT6, [46]) from the RCSB database. The lipids (DDPC— dipalmitoylphosphatidylcholine and DSM—dihydrosphingomyelin) were modeled using CHARMM-GUI, and the structure was available under this user interface. Water was modeled using TIP3P. The remaining space in the simulation box was filled with explicit water molecules. In addition, K^+^ and Cl^−^ ions were introduced into the system to ensure charge neutrality.

A CHARMM 36 m force field [47] was used to conduct the MD (molecular dynamics) simulation. Additional details on the fundamental form of the CHARMM force field [48] are available in Appendix A.

**Figure 2:**
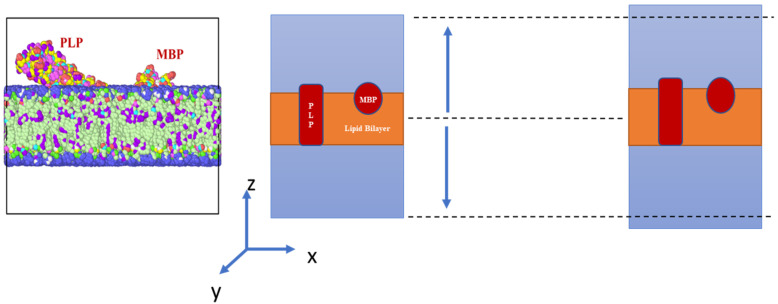
Schematic representation of the tensile test scheme for the representative element (Dotted lines representing the original frame of reference).

To simulate the tensile response of our myelin model, a constant tensile strain was applied to the simulation model along the thickness direction (z-direction). To ensure uniform tensile loading, the uniform strain load was exerted individually to every atom of the simulation model. Tensile loading was initially imposed at an increment of 5 percent. When failure began, a shorter increment (i.e., 1 percent) was used. The test scheme is illustrated in Figure 2, as follows:

The process (Figure 2) was repeated for subsequent increments to generate a complete stress strain response. To conduct the simulation, at first the simulation model was minimized at a temperature of 310 K, considering the human body temperature averages 37 °C. The energy equilibration was carried out under an isothermal–isobaric ensemble (NPT) for a total of 1 ns (nanoseconds), at a goal pressure of 1 bar. The relaxation rate (W) for the system during equilibration was governed by Equation (1):

(1) $$W=\left(N\;+\;1\right)K_{b}T_{target}P_{damp}^{2}$$

where $K_{b}$ is the Boltzmann constant, N is the total number of atoms, $P_{damp}$ is the time units at which the pressure is relaxed and $T_{target}$ is the target temperature. At the end of equilibration, it is expected that the system will be fully equilibrated and will remain in a stress-free state. A constant tensile strain of 5 percent was applied in increments to the system in a z-direction at an interval of 50 Ps, and periodic boundary conditions were maintained throughout the simulation. The time step for our simulation scheme was 1 fs (femtoseconds).

The stress strain response was determined following the method adopted in a recent publication investigating the mechanical behavior of actin and spectrin [49]. Using LAMMPS [50], embedded in a supercomputer stampede2 of TACC (Texas Advanced Computing Center, Austin, TX, USA), the per atomic stress tensor was calculated in the form of stress × volume. The per atom stress calculation is governed by the following equation in LAMMPS:

(2) $$\begin{array}{ll} W_{ab}=\frac{1}{2}\sum_{n=1}^{N_{p}}(r_{1a}F_{1b} & +r_{2a}F_{2b})+\frac{1}{2}\sum_{n=1}^{N_{b}}(r_{1a}F_{1b}+r_{2a}F_{2b})+\frac{1}{3}\sum_{n=1}^{N_{a}}(r_{1a}F_{1a}+r_{2a}F_{2b}+r_{3a}F_{3b})+\frac{1}{4}\sum_{n=1}^{N_{d}}(r_{1a}F_{1a}\\ & +r_{2a}F_{2b}+r_{3a}F_{3b}+r_{4a}F_{4b})+\frac{1}{4}\sum_{n=1}^{N_{i}}(r_{1a}F_{1a}+r_{2a}F_{2b}+r_{3a}F_{3b}+r_{4a}F_{4b})+Kspace\left(r_{ia}F_{ib}\right)\\ & +\sum_{n=1}^{N_{f}}r_{ia}F_{ib} \end{array}$$

The virial contribution is the summation of pairwise interactions for $N_{P}$ neighbors, $N_{b}$ bonds, $N_{a}$ angles and $N_{d}$ dihedral and $N_{i}$ improper contributions. There is an additional kspace term for long-range coulombic interactions, and finally the last term applies for the fixes ($N_{P})$ that have been imposed on the system. These parameters are defined by CHARMM-GUI.

The volume was extracted by utilizing the Voronoi feature in LAMMPS. Following that, the pure stress values were obtained from the calculated stress tensor. During the tensile test, the NVT ensemble was maintained with a damping value of 100 fs. The average stress values, corresponding to the imposed strain, were calculated and plotted. The image snapshots were obtained from OVITO visualization software (The version number is 3.5.3) [51].

The following set of governing Equations (3)–(8), as represented in matrix form, was used to determine the values of the elastic modulus (E) and Poisson’s ratio (ϑ) of our myelin model.

(3) $$\left(\begin{array}{c} \sigma_{xx}\\ \sigma_{yy}\\ \sigma_{zz}\\ \tau_{xy}\\ \tau_{yz}\\ \tau_{zx} \end{array}\right)=\left(\begin{array}{cccccc} \hat{E}(1-\vartheta ) & \hat{E}\vartheta & \hat{E}\vartheta & 0 & 0 & 0\\ \hat{E}\vartheta & \hat{E}(1-\vartheta ) & \hat{E}\vartheta & 0 & 0 & 0\\ \hat{E}\vartheta & \hat{E}\vartheta & \hat{E}(1-\vartheta ) & 0 & 0 & 0\\ 0 & 0 & 0 & G & 0 & 0\\ 0 & 0 & 0 & 0 & G & 0\\ 0 & 0 & 0 & 0 & 0 & G \end{array}\right)\left(\begin{array}{c} \epsilon_{xx}\\ \epsilon_{yy}\\ \epsilon_{zz}\\ \gamma_{xy}\\ \gamma_{yz}\\ \gamma_{zx} \end{array}\right)$$

(4) $$\hat{E}=\frac{E}{\left(1-2\vartheta )(1-\vartheta \right)}$$

(5) $$\sigma_{ij}^{V}=\frac{1}{V}\sum_{\alpha }[\frac{1}{2}\sum_{\beta =1}^{N}(R_{i}^{\beta }-R_{i}^{\alpha })F_{j}^{\alpha \beta }-m^{\alpha }v_{i}^{\alpha }v_{j}^{\alpha }]$$

where $\sigma_{ii}$ represents the normal stresses in the i^th^ direction (i = x, y, z), which is based on the virial stress formulation $\sigma_{ij}^{V}$ [52], E is the Young’s modulus, G is the shear modulus, $\tau_{ii}$ represents the directional value of shear stresses, ϑ is the Poisson’s ratio, ϵ is the normal strain and γ is the shear strain, respectively. The directional values of stresses ($\sigma_{ii})$ during each increment were obtained from the molecular dynamics simulation under a constant stretched condition. For the virial stress ($\sigma_{ij}^{V})$ formulation, (i,j) represents the x,y,z directions, β represents N neighbors of atom α, $R_{i}^{\alpha }$ is the position of the atom α in the i-th direction, $F_{j}^{\alpha \beta }$ is the force in the direction j on the atom α due to atom β, V is the total volume, $m^{\alpha }$ is the mass of atom α and $v^{\alpha }$ is the thermal excitation velocity of atom α. For extracting the value of pure stresses, we used original volume ($V_{0})$ in place of instantaneous volume (V), as our analysis included engineering stresses not true stresses.

For our loading condition,

(6) $$\left(\begin{array}{c} \epsilon_{xx}\\ \epsilon_{yy}\\ \epsilon_{zz}\\ \gamma_{xy}\\ \gamma_{yz}\\ \gamma_{zx} \end{array}\right)=\left(\begin{array}{c} 0\\ 0\\ \epsilon_{zz}\\ 0\\ 0\\ 0 \end{array}\right)$$

Therefore, the corresponding stress states can be found as long as the linear elastic stress–strain relation is maintained. The elastic modulus (E) and Poisson’s ratio (ϑ) of myelin can then be obtained as:

(7) $$\vartheta =\frac{\sigma_{xx}}{{\sigma_{zz}+\sigma }_{xx}}$$

and

(8) $$E=\frac{\sigma_{zz}(1-2\vartheta )}{\epsilon_{zz}}$$

## 3. Results

### 3.1. Stress Strain Response for the Representative Model

**Figure 3:**
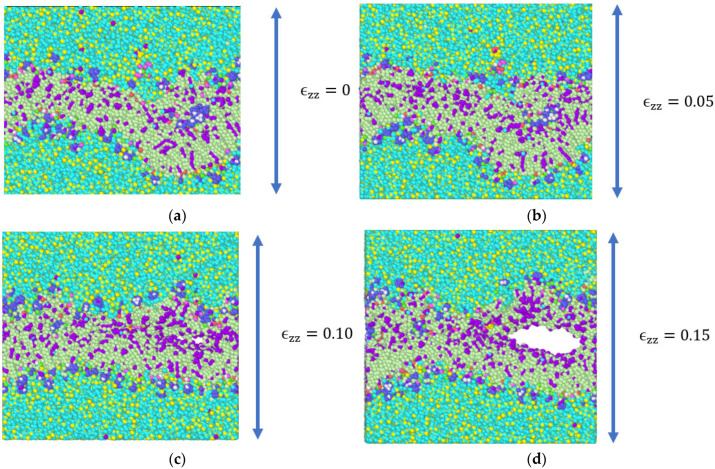

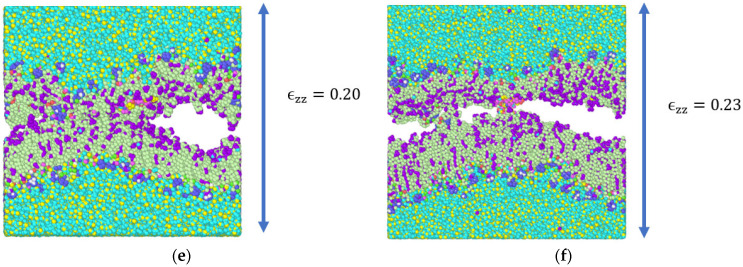
Snapshots of the simulation model taken at the end of equilibration after the application of constant tensile strain in increments (arrows indicate the application of strain along the thickness direction). (**a**) Equilibrated model without any strain on the system; in (**b**–**e**) 5 percent strain was applied in increment at every step; and in (**f**) 3 percent increment in the tensile strain resulted in final failure.

**Figure 4:**
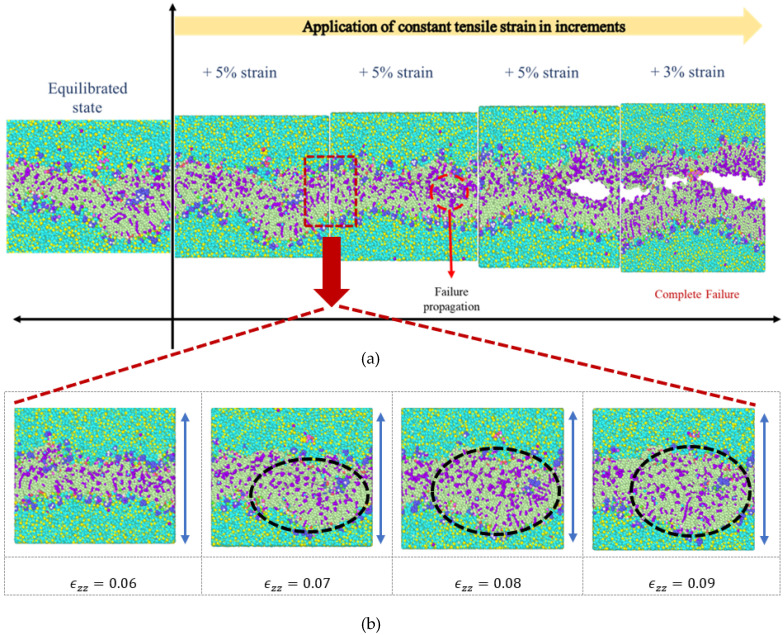
(**a**) Snapshots of the system from the equilibrated state to the application of constant strain in increments. (**b**) Snapshots of the simulation model between 5% and 10% constant tensile strain application, in increments of 1 percent.

**Figure 5:**
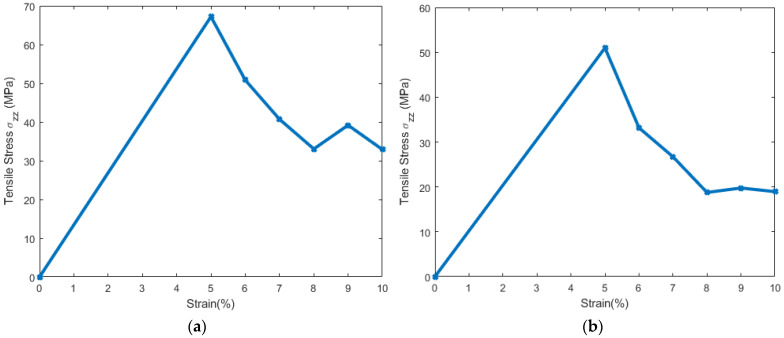
(**a**) Average stress ($\sigma_{zz})$ vs strain (%) for the entire simulation model. (**b**) Average stress ($\sigma_{zz})$ vs. strain (%) for the bilayer and protein in the system.

A constant tensile strain of 5 percent was applied in increments to the simulation model in the z-direction (Figure 2) at each step and the average engineering stresses respective to every applied strain were computed. Figure 3 and Figure 4 show the snapshots for the simulation model due to the imposed tensile strain. From the figures, it is clear that failure began in the bilayer within the system right after the 5 percent strain. Total failure was observed when the model was stretched beyond 23 percent tensile strain. The average engineering stresses ($\sigma_{zz}$) along the direction of tensile loading were plotted against strain (%) (Figure 5a) and from the plot the observance of peak stress at 5% strain reveals the fact that initial failure began between 5% and 10% strain. From Figure 3 and Figure 4, it can be observed that the failure was initiated within the system at around 5 percent and was propagated to the final failure point. To further examine the failure in between peak stress (5 percent) and failure propagation (10 percent), a shorter increment (1 percent) of tensile strain was applied within this range. From the snapshots (Figure 4b), it can be observed that the bilayer separated (as marked by dotted lines) with a gradual increment in tensile loading.

### 3.2. Stress Strain Response for the Bilayer and Protein

From the snapshot representation (Figure 3 and Figure 4), it can be observed that failure was initiated in the bilayer. As such, we have also estimated the average stress ($\sigma_{zz}$) developed in the bilayer with respect to the imposed incremental constant strains (Figure 5b) for the bilayer. Using the peak value of stresses, the modulus of elasticity and the Poisson’s ratio were calculated using Equations (7) and (8) and are tabulated in Table 2.

**Figure 6:**
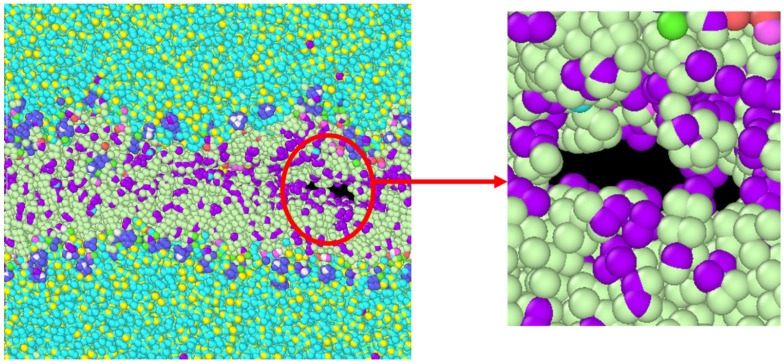
Detailed view of the failure initiation of the system at 10 percent strain application in the z-direction.

**Figure 7:**
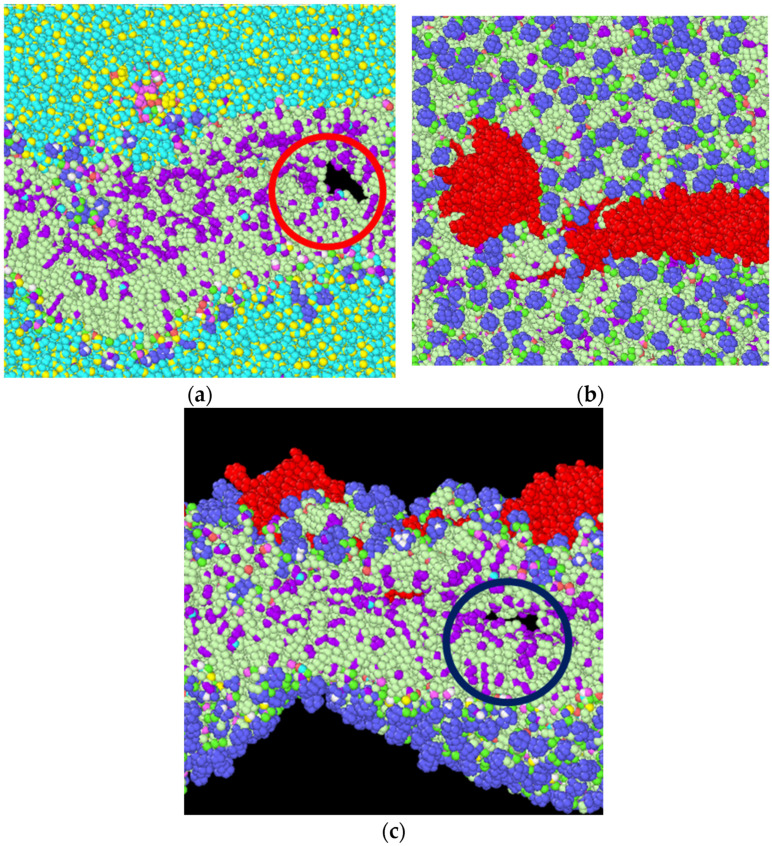
(**a**) The red circle shows the failure initiation within the bilayer at 10 percent strain. (**b**) The top view of the system, showing the undisturbed structure of proteins (marked in red), with MBP (smaller structure, left) and PLP (long structure, right). (**c**) The side view, similar to Figure 7a, shown by removing water and ions for visual clarity. It shows that the flaw generates within the bilayer (marked in blue circle), not in the proteins.

From the results of constant strain application to our simulated model, we observed that, with the gradual increment in loading, the failures were initiated and propagated to the final failure point within the bilayer, with no failure initiation within the protein structures. The incompressible nature of the system can be depicted from the values of Poisson’s ratio for the entire system and the system components, as tabulated in Table 2. A detailed representation of flaw generation within the bilayers has been depicted in Figure 6, and concurrently, Figure 7 represents the structure and the behavior of the proteins during failure initiation.

## 4. Discussion

In our study, we attempted to address the injury criterion at a sub-cellular level through computation. The first part of our computation involved developing a representative model of the myelin layer. In the second part of our computation, we evaluated the tensile strain (and stress) limit of the myelin layer. We believe that our findings will benefit the scientific community interested in finding the strain-based cellular level TBI injury risk curve. Considering the peak stress at the maximum tolerable strain, we evaluated the Poisson’s ratio and elastic modulus at the sub-cellular level. We would like to add a caution that the tensile stress limit estimated at the molecular level needs careful consideration, as the direct relations between molecular-level stresses and continuum-level stresses are not often well defined. As such, the strain limit for myelin is a more suitable measure for the cellular-level injury limit.

The results of our simulation reveal that failure was initiated at approximately 10 percent strain application. The further application of tensile stretch led to damage propagation.

In previous works in the literature [40,41,42], it was observed that myelin is built on multiple rolls of lipid bilayers wrapped over an axon. Each layer is connected to the others through MBP. The transmembrane protein (PLP) is embedded in between each myelin layer to maintain the chemical balance within the layers. Currently, there are no solved structures for MBP and PLP in the protein bank. For our simulation model, we selected 4BVM for MBP, a combination of P2 fatty acid binding protein and MBP, which is responsible for the anchoring of two adjacent lipid bilayers [45]. For the transmembrane protein, there was a predicted alpha fold structure [53]. Based on the limited evidence and supporting information, the protein structure that closely related to the functionality of the transmembrane protein was 1WT6 [54].

Through our study, we aimed to assess the failure criteria for the unit representing the layer of the myelin sheath. To understand how the system would respond to sudden tensile force, we applied a constant strain incrementally to the system. As mentioned earlier, we observed a failure initiation at approximately 10 percent strain (Figure 5). A closer view (Figure 6) of the failure at 10 percent strain reveals that the failure continued closer to the edges of the system within the lipid bilayer. A representation of the protein structures at the failure point shows the undisturbed structures of the protein during failure initiation (Figure 7). Additionally, the value of the tensile stress on the protein structure, immediately before the system degenerated, can be glimpsed from Table 3. This value of stress has limitations, as the correlation between molecular-level stress and continuum-level stress is not well defined.

**Figure 8:**
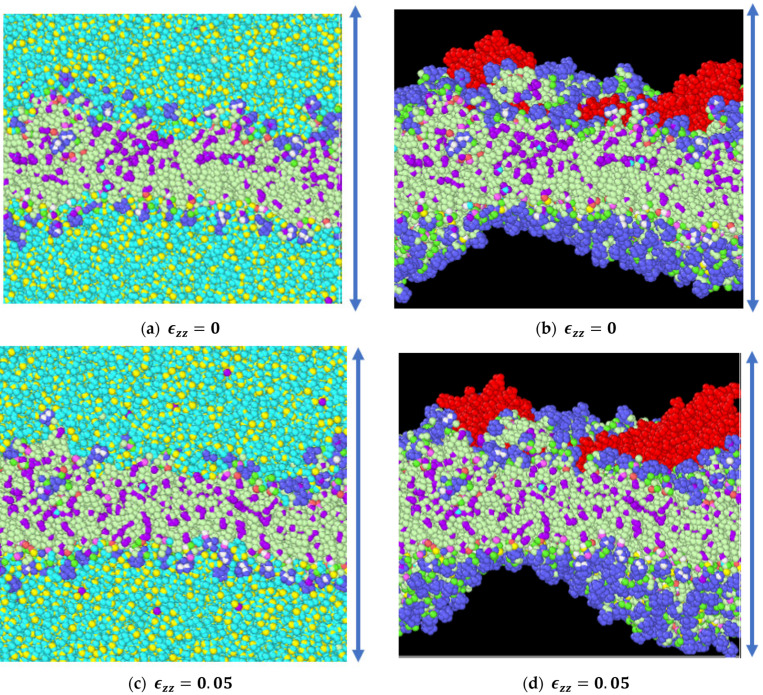
(**a**) System without any strain at the end of equilibration in NVT (for a duration of 250 ps (picosecond)). (**b**) Unstrained system at the end of equilibration, where waters and ions have been removed for visual clarity and proteins are marked in red. (**c**) System with 5 percent strain at the end of equilibration in NVT (for a duration of 200 ps (picosecond)). (**d**) System with 5 percent strain at the end of equilibration, where waters and ions have been removed for visual clarity and proteins are marked in red.

**Figure 9:**
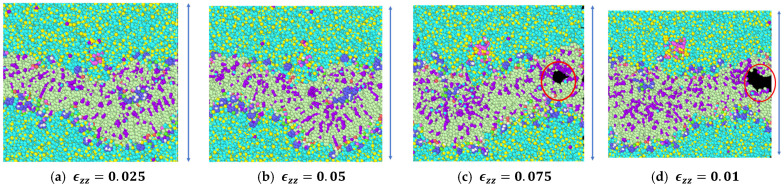
Strain application along the thickness of the model at 2.5 percent increments along the z-direction; the red circle marked shows the failure within the model.

As observed from our simulations, the application of 5 percent tensile strain was the first control point and flaw generation appeared in the system immediately after this point, i.e., between 5 percent and 10 percent strain. In order to validate the presence of system instabilities, we allowed our simulation model to equilibrate, in the absence of strain, under the same conditions for the time until the final failure point. It was observed that during this equilibration period there were no failures within the system. Additionally, we carried out equilibration simulations with 5 percent strain application to verify the presence of system instabilities (Figure 8). Also, simulations were carried out with 2.5 percent tensile strain increments and, as in our first observation, defects were observed in the model past 5 percent tensile strain application, i.e., our second control point (Figure 9). Additional simulation snapshots and energy history is provided in Appendix B, to clarify whether system instabilities were present or whether such instabilities played any role on the final outcome.

From Figure 3 and Figure 4, it was also observed that the failure started at around 10 percent strain and the system completely separated at around 23 percent strain. Although failure was initiated at approximately 10 percent strain, we continued our strain application until the final failure point. This was because for any complicated system at the molecular level, there is a probable chance of molecular rearrangement and reattachment. It was important to address this phenomenon within our system. During each increment of strain application, it was observed that there was a molecular rearrangement and relaxation to release the stress experienced due to tension. As such, it appears that the loading applied to the system at each step was long enough to release the stress before the next loading was applied.

This behavior of the system can be explained by the calculated value of Poisson’s ratio (Table 2). It can be observed from the tabulated values that for the entire system (water and ions included) and for the lipid bilayer and protein (excluding water and ions), the value was close to 0.5.

Thus, it can be stated that the physical state of the single myelin is nearly incompressible. Using Equations (3)–(8) and the recorded initial stress strain response, the modulus of elasticity (E) and Poisson’s ratio (ϑ) of the system were obtained and are shown in Table 2. It is noted that the stress values were obtained using the per-atom stress tensor from LAMMPS, and to obtain the true stress values, the Voronoi feature was utilized. For our study, we considered engineering stress instead of true stress. The average stresses corresponding to respective applied strains were recorded and used in our calculation.

One important aspect of the myelin sheath is the presence of water layers around it. Therefore, an important aspect that can be addressed in detail in upcoming studies is the maximum thickness of the hydration layer. It is estimated that the bond separation of different atoms lies in the range of 0.7–1 nm. These specific values can be determined with the help of the LJ (Lennard-Jones) potential [55,56,57]. Based on the aforesaid values, a maximum value of 1 nm was preferred for the water thickness.

Using scanning force microscopy (SFM), the failures of single- and multilayered lipid bilayers were studied by varying the number of chain lengths, the lamellae and the head group composition. Implementing the optimized pulse force mode, the results obtained for the adhesion force and stiffness of the membrane suggested different failure patterns depending on the lipid type [58]. Electron micrograph images showed how demyelination and remyelination followed post-mild TBI (traumatic brain injury). It was also observed that, during remyelination, myelin figures do not wrap around the axons in a spiral structure; rather they take the form of a thread-like long structure, suggesting the disruption of compactness post trauma [16]. Results, found using an in vivo test device, support the formation of long thread-like remyelinated figures. It was observed that mechanical stretch activated extracellular signal-regulated kinase (Erk1/2) following intracellular Ca^2+^ activation, but the mechanisms by which this takes place were unclear from these findings. This phenomenon led to myelin protein (MBP) loss, and in absence of MBP the compactness of the myelin sheath was compromised [59]. Furthermore, results from an experimental rodent model for multiple sclerosis also suggest the degeneration of myelin in different strains, by evaluating the MBP loss in different variants of mice over time [60].

Earlier studies reported a threshold value for strain in the case of diffuse axonal injury (DAI). In this study, the influence of brain mass and rotational acceleration was taken into consideration and, with the help of physical and analytical models, it was confirmed that the critical shear strain for moderate to severe DAI ranged between 5 and 10% [61]. This value was in close agreement to what we attained from our computational study. Again, finite element modeling of the human brain with accident reconstruction portrayed the onset of concussion in various regions of the human brain, and a 50 percent concussion probability within the corpus callosum at 0.21 strain was observed [62]. Several in vivo and in vitro animal model studies exist related to traumatic brain injury. Findings related to nanoparticle transportation across the blood–brain barrier (BBB) post-controlled cortical impact provide insights into the BBB post TBI [63]. The influence of organic solvents on secondary brain damage has been studied during experimental TBI to evaluate the neuroprotective behavior [64]. From analyzing postmortem human brain tissue samples, the influence of inflammatory proteins in microglia, astrocytes and neurons was found. This study highlighted the factors that are responsible for pyroptosis pathways during Alzheimer’s disease [65].

It has been observed, from recent experimental and computational studies, that myelin damage can be connected to the pathological pathways for neurodegenerative disorders. Information and evidence related to the mechanical aspects of brain trauma are lacking. Our research aims to provide a concise and clear idea about the injury thresholds of brain damage at a sub-cellular level. Myelin, as the protective covering of the axon, plays an important role in signal transmission and the integrity of neuronal cell health.

## 5. Conclusions

In summary, through our study we attempted to discover the mechanical response of a single myelin layer under tensile loading. We applied incremental tensile loads, and the mechanical response was assessed from the stress strain response. It was observed that the stress value dropped as soon as failure was initiated within the system. The mechanical behavior of the representative myelin layer was evaluated by determining ϑ and E. As mentioned earlier, myelin is basically a roll of lipid bilayers stacked on top of one another. As the overall mechanical response of the myelin sheath is governed by the interfacial properties in between the lipid bilayers, as well as the cohesive properties of the myelin layer, this study was limited to the cohesive response of a single myelin layer. The interfacial properties of the myelin–myelin layer will be considered in our future research, to give a comprehensive understanding of the mechanical properties of the myelin sheath.

## Figures and Tables

**Table 1:** Single myelin model constituent composition by percentage.

Constituent	Composition (%)
Water	61.24
Lipid	36.50
MBP (myelin basic protein)	0.86
PLP (proteolipid protein)	1.28
Ions (K^+^, Cl^−^)	0.12

**Table 2:** Modulus of elasticity and Poisson’s ratio.

Component	Modulus of Elasticity (E) MPa	Poisson’s Ration (ϑ)
Total system	70.86	0.491
Lipid bilayer and protein	24.21	0.496

**Table 3:** Average tensile stress on the protein structures due to strain application.

Component	Average Tensile Stress ($\sigma_{zz}$) MPa
PLP	63.09
MBP	64.44

**Figure A1:**
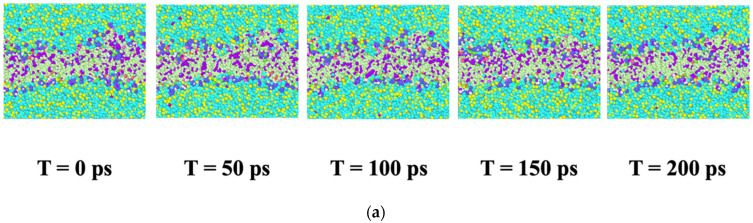

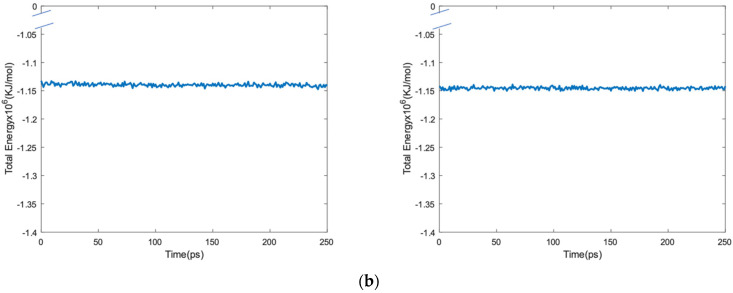
(**a**) MD snapshots taken at 50 ps intervals during the long-time equilibration of the simulation box at 0% strain state. (**b**) Total energy vs. time plot: the left plot shows the response of our original simulation cell and the right one is the newly studied independent cell. It is evident that the system remains stable at the unloaded state.

**Figure A2:**
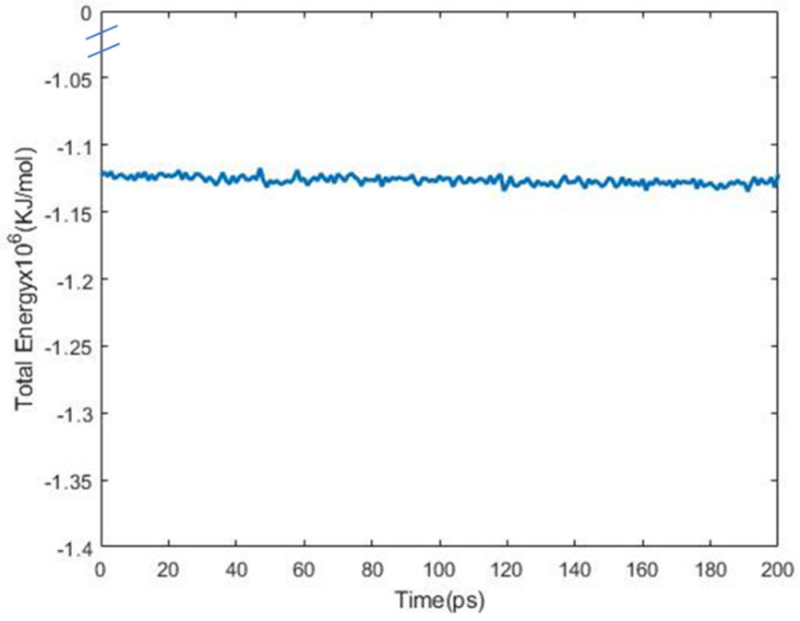
Total energy vs. time plot during equilibration for the system hold at 5% tensile strain.

**Figure A3:**
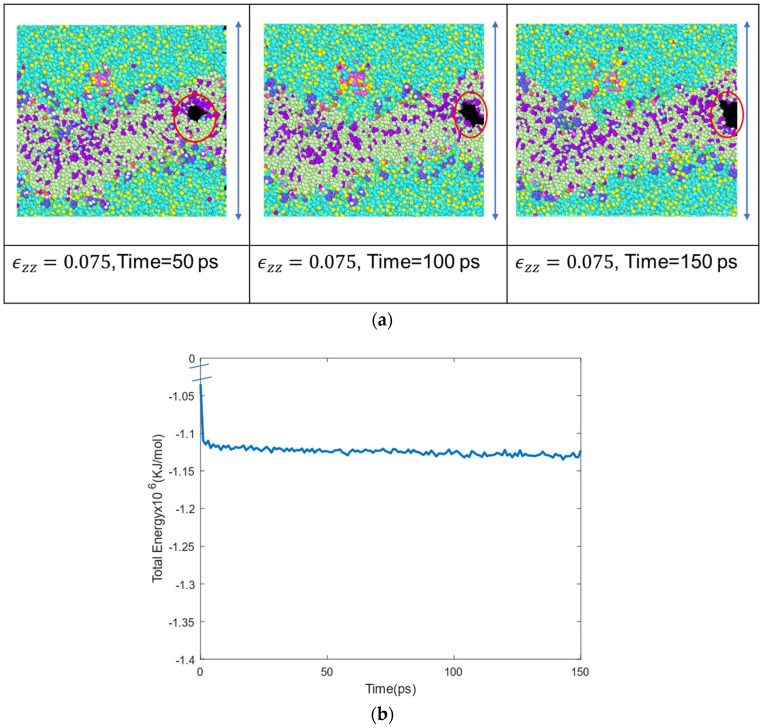
(**a**) MD snapshots taken at 50 ps intervals (red circles indicate failure) for system strained at 7.5% and equilibrated in that strained state under NVT for 150 ps. (**b**) Total energy curve for system strained at 7.5% and equilibrated under NVT for a long time.

## Data Availability

All data, including raw data, simulation input files, post-processed data, results and charts, are stored on a local cloud server. Data can be made available upon request.

## Appendix A

The fundamental form of the CHARMM force field is as follows:

(A1) $$E_{total}=E_{bonded}+E_{bonded}$$

(A2) $$E_{bonded}=\sum_{bonds}K_{b}{\left(b-b_{o}\right)}^{2}+\sum_{angles}K_{\theta }{\left(\theta -\theta_{o}\right)}^{2}+\sum_{\begin{array}{c} improper\\ dihedrals \end{array}}K_{\varphi }{\left(\varphi -\varphi_{o}\right)}^{2}\;+\sum_{dihedrals}\sum_{n=1}^{6}K_{\phi ,n}\left(1+\cos\left(n\phi -\delta_{n}\right)\right)+\sum_{Urey-Bradley}K_{UB}{\left(r_{1,3}-r_{1,3;0}\right)}^{2}$$

(A3) $$E_{non-bonded}=\sum_{non-bonded\;pairs\;i,j}\frac{q_{i}q_{j}}{4\pi D\|r_{i}-r_{j}\|}+\sum_{non-bonded\;pairs\;i,j}\varepsilon_{ij}[{\left(\frac{R_{\min i,j}}{\left\|r_{i}-r_{j}\right\|}\right)}^{12}-{\left(\frac{R_{\min i,j}}{\left\|r_{i}-r_{j}\right\|}\right)}^{6}]$$

The CHARMM force field comprises bonded and non-bonded interactions. The bonded interactions include bonds, angles, improper, dihedrals and an additional Urey-Bradley term. The non-bonded term comprises the interactions due to electrostatic and van der Waals forces. In Equation (A2), the subscripts b, θ, φ and ϕ represent the bond length, valance angles, improper dihedral angles and dihedral angles, respectively, that are determined from the molecular geometry, and $b_{o}$, $\theta_{o}$ and $\varphi_{o}$ represent the corresponding equilibrium values. The force constants for bonds, angles and improper dihedrals are denoted by $K_{b}$, $K_{\theta }$ and $K_{\varphi }$. For the dihedral term, $K_{\phi ,n}$ represents the amplitude and $\delta_{n}$ is the phase angle. The additional Urey-Bradley term indicates the cross term due to terminal atom interactions that is not accounted for by the bonds and angles, where $K_{UB}$ indicates the force constant, $r_{1,3}$ indicates the distance between the terminal atoms and $r_{1,3;0}$ is the equilibrated distance for the terminal atoms. The additional term provides refinement in the vibrational modes of the reference compounds. In the case of the non-bonded term, D denotes the electrostatic term, $q_{i}{,q}_{j}$ are the partial atomic charges, $\varepsilon_{ij}$ is the well depth and $\left\|r_{i}-r_{j}\right\|$ defines the distance between the atoms i and j, respectively. The specific parameters for different molecules were inbuilt in the CHARMM-GUI.

## Appendix B

To address whether there exists any internal instability or whether the presence of such instability influences the outcome, such that the reproducibility of simulations becomes questionable, we took two measures. First, we allowed our system to equilibrate at 0% applied strain for 200 ps in NVT and observed the molecular structure at every 50 ps. We also monitored the total energy history as the simulation continued. Figure A1 reports the result. It is evident from Figure A1 that our system remains stable, and no flaw formation is observed on the unloaded model during equilibration. Second, we equilibrated the system after 5% strain is applied. Note that the system failed at around 7.5% strain. Figure A2 shows the energy history of the system equilibrated while stretched at 5% tensile strain. It is evident that, in the absence of additional applied strain beyond 5%, the system remains stable at the state. In both cases, we observed no failure initiation in the system. In summary, we can confirm that incremental external force is required to enable crack formation and growth and that there is no evidence of system instabilities.

To check whether a crack can grow spontaneously even if we stop loading the cell further, we allowed our system to equilibrate under the same conditions but in the absence of strain for a substantial amount of time (up to 150 ps). As shown in Figure A3, the system remained at that state, and there is no evidence of spontaneous crack growth because of system dynamics.
